# The influence of education on the access to childhood immunization: the case of Spain

**DOI:** 10.1186/s12889-018-5810-1

**Published:** 2018-07-18

**Authors:** T. Mora, M. Trapero-Bertran

**Affiliations:** Research Institute for Evaluation and Public Policies (IRAPP), Universitat Internacional de Catalunya (UIC), Immaculada 22, 08017 Barcelona, Spain

**Keywords:** Income gradient, Preventive care, children’s health, Education

## Abstract

**Background:**

In order to enhance childhood vaccination uptake and the health consequences for the whole society, there is a need to study predictors that might help in understanding parents’ behaviour in relation to childhood vaccination schemes. The aim of this paper is to assess whether parental education has an influence on their children’s public health-care use in terms of visits for vaccinations, and thus evaluate whether more educated parents use public health resources more frequently in childhood immunization schedules.

**Methods:**

The setting was the region of Catalonia in the north-east of Spain. Three different databases, containing information about 11,415 individuals corresponding to 79,905 observations, were merged and linked: 1) observational and longitudinal administrative data for adults and children in Catalonia; 2) a database containing information on the vaccination of children in relation to the public health programme called the “Healthy Child Programme”; and 3) the governmental vaccination registration. The presence of an education gradient was explored using a logistic regression. Children’s health-care use was modelled using a logistic procedure.

**Results:**

The greater the mothers’ educational attainment level, the higher the probability of being vaccinated in this immunization programme. The presence of an age profile for vaccinations showed that less educated parents visit their GPs more frequently for immunizations when their children are below the age of six, but that pattern is completely the opposite after that age. Hence, for children aged between six and 16, more educated parents are more likely to ensure their children are immunized. Likewise, systematic vaccinations are more likely for those parents with a lower educational attainment level.

**Conclusions:**

This paper evidenced the presence of an education gradient for specific preventive care through the public health system and visits to the GP without any particular disease or advice for specific vaccinations.

## Background

Vaccinations are one of the most important tools of primary prevention. All countries in the European Union (EU) have a long tradition of implementing vaccination programmes [1–3]. Vaccination is a safe and cost-effective way to protect people – especially infants and young children – from certain infectious diseases [4–8]. The annual return on investment in vaccination has been calculated to be between 12 and 18% [9].

All EU countries have a “vaccination schedule”, recommending that the vaccines be given at various ages during childhood. In Europe, childhood immunization programmes have been instrumental in controlling infectious diseases, but too many children in Europe go unvaccinated and remain vulnerable to potentially life-threatening diseases [8].

The non-systematic immunizations from birth in Spain, according to the European Centre for Disease Prevention and Control (ECDC) and the Immunization Calendar from the Spanish Association of Paediatrics (SAP), are: diphtheria, tetanus, pertussis, poliomyelitis, haemophilus influenzae type b infection, hepatitis B, pneumococcal disease, meningococcal disease, measles, mumps, rubella, varicella, human papillomavirus infection, and influenza. In Catalonia, the same guidelines are followed but they are all classified into two categories: a) systematic vaccinations, those which are provided by the national system and are recommended (diphtheria; tetanus; pertussis; poliomyelitis; haemophilus influenzae type b infection; hepatitis B; pneumococcal disease; meningococcal disease; measles; mumps; rubella; varicella; and human papillomavirus infection); and b) non-systematic vaccinations, those which are not provided by the national system (rotavirus and chickenpox), and are recommended and voluntary. There are non-systematic vaccinations specifically for risk groups (influenza and hepatitis A), which are recommended only to those that, because of their characteristics, are at greater risk of having a disease or the disease worsening if they are not vaccinated. In fact, some of these non-systematic vaccinations, such as pneumococcal, rotavirus, and human papillomavirus vaccinations, have been controversial in terms of their effectiveness given that intense debates have taken place in the media and among physicians [10]. In Catalonia a great part of the population has double health care coverage, public and private, most in the privileged population. This might influence the conclusions of this study.

In order to enhance childhood vaccination uptake and the health consequences for the whole society, there is a need to study predictors that might help in understanding parents’ behavior in relation to childhood vaccination schemes. Resources, as a predictor, have previously been used in different ways in order to evaluate the influence of different variables on childhood immunization schedules. For instance, the effect of parental research on health-care use has previously been studied [11]. Differences in health according to education are also documented [12]. With regard to the effects of parental education on children’s health, the most common finding is that after including parental education in regression analyses, income effects considerably decrease in magnitude [13]. However, two exceptions are worthy of mention. Evidence for the UK showed that neither maternal nor paternal education has any association with child health [14] whereas other evidence found that income has a strong independent effect after controlling for educational attainment levels and unemployment [15]. In order to discover the effect of parental educational levels on children’s health status, some papers used “natural experiments” exploiting exogenous variation in education induced by school reforms [16] or using adoptees’ data sets to separate nurturing from nature effects [17].

As far as we know, little research has been conducted in this regard using real data on the use of resources. Indeed, a recent systematic review has been published regarding the factors associated with incomplete or delayed vaccination across countries [18]. These authors concluded that strengthening the contacts and relationships between the health-care services and mothers with several children and families with a low educational level/low socio-economic status appears to be an important step in improving vaccination coverage. In addition, previous work has been published indicating that socio-economic status is a useful tool for vaccine delivery research among children [19].

On the one hand, a greater number of visits for advice about vaccinations can occur mainly because the greater the parental education, the more able parents are to identify severe ill health, and thus, the more conscious they are of the need to visit a doctor [20]. Likewise, conditional on visiting a GP, more educated parents might be more likely to become more convinced about the importance of systematic and controversial vaccinations [21]. On the other hand, a negative answer is expected a priori given that more educated mothers are more likely to be enrolled in the labour market, and, as a consequence, experience more time constraints, although they could experience lower differential penalties for missing work. Another hypothesis would consist of the expected negative effect arising from more educated parents more consciously identifying what really requires a new visit to a GP or specialist. The novel contribution of this study was to generate evidence using more observational and longitudinal administrative data than previous literature. To the best of our knowledge, no previous literature has tackled the likelihood of children being vaccinated being conditional on parental socio-economic status (SES), and particularly education measured using counts of vaccination units. In order to do so, information should be linked regarding sociodemographic characteristics and visits to the GP, visits for a specific children’s vaccination programme, the vaccination received by the children, and levels of education and income of children’s parents. Therefore, the aim of this paper was to assess whether parental education has an influence on childhood immunization schedules, measured in terms of visits for vaccinations, and evaluating whether more educated parents use public health resources more frequently.

## Methods

The study comprised an analysis of cross-sectional data, for all the screenings made for the period between 2004 and 2012, for the whole population of children and their parents located in the region of Catalonia in the north-east of Spain. The primary outcomes of the analysis were the likelihood of being vaccinated and the number of vaccinations in relation to the schooling years of the children’s parents. The secondary outcomes were visits for advice about systematic and controversial vaccinations. All counts were estimated by means of negative binomial regressions and, for those variables indicating the likelihood of children being vaccinated in different age categories, logistic regressions were used. Relevant influences are summarized by the following reduced-form equation:1

where H denotes health status and B represents individual behaviour, lifestyle while the subscript c means children, p denotes parental, and t indicates the analysed time period *t* = 2004…2010. The most important reason for using the health service was a person’s need. Thus, health status degree was proxied through a list of dummy variables representing children experiencing severe diseases (hypertension, lipid and cholesterol problems, cardio problems, bronchial asthma and chronic obstructive pulmonary disease, the presence of any mental disorder and malignant neoplasms). The Charlson index, which categorizes co-morbidities of patients based on diagnosis codes, was also included. With regard to health-care provision, the distance (D) between children’s residence and the closest GP, i.e. the one they belong to, was also computed for both hospitals involved in kilometres through the use of individual residential location and health-care facility location. Parental behaviour (Bp), lifestyle was captured through lifestyle habits (BMI and dummies representing smoking and heavy alcohol drinking), while X contains a list of covariates denoting differences in sociodemographic characteristics (age, female, status of immigrant) or being the firstborn in the household. Children’s health-care use (HCU) was modelled by means of individual determinants and the health-care system characteristics. Parental education was assessed using schooling years. All these variables are regressors introduced additively. We also controlled whether any of their parents had deceased. Marginal effects were reported.

In order to conduct this analysis, the data used were collected from four different databases and they were merged and linked by means of personal confidential information. The linking process was carried out by the Statistical Institute of Catalonia (IDESCAT), who made sure that researchers did not have access to any confidential information. The analysis was started when the Ethical Research Committee (CER) at the Universitat Internacional de Catalunya (UIC) gave its ethical approval for this study. The data from the three databases were confidential, implying that all materials were secure and that no material obtained from the Standards Database was disseminated or otherwise provided to any person not currently an authorized user. The first three databases were originated by the same population, and contained observational and longitudinal administrative and medical records until individuals were 16 years old followed up over seven consecutive years in six primary care centres and two reference hospitals, comprising seven health districts. These centres served more than 110,000 inhabitants in the north-eastern area of Barcelona, Catalonia. The study also considered those who deceased during the analysed period. All these register data were additionally merged, through a unique identifier, into a fourth database corresponding to the population census, which allowed us to incorporate new variables for each patient (e.g. education, marital status and employment status) that were not available in the original sample, although the last Spanish census was conducted in 2001. The information we matched corresponded to that specific year. Educational information was obtained at nine levels, although these data were transformed into schooling years for an easier interpretation.

The first database incorporates a rich set of information regarding the utilization of health-care resources (number of visits to the GP, specialist and emergency care), and sociodemographic characteristics such as the patient’s age, gender, employment status (active/retired), place of birth and habitual residence. These variables were used as a control in all regressions. The second database contained information on children’s vaccination in relation to the public health programme called the “Healthy Child Programme” for preventive care. This programme, funded by the government in Catalonia, encourages actions to promote health and prevention in the paediatric age range. Using this database, the authors expected to evidence a higher probability of health-care use by less educated parents given that the implementation of the programme promoted calls to increase the attendance of children at greater risk. The influence of education on the “Healthy Child Programme” was assessed through the number of visits for advice about systematic vaccinations and non-systematic controversial vaccinations. Given that vaccinations should be paid for supplementary when parents bring their children to private consultations, some of them might attend the public health system to obtain this preventive care for free. The third database contained governmental vaccination registration. This database consisted of administrative registers containing all provided vaccinations, and was thus a register of all vaccinations for children in the public health system. Free immunizations were amongst the highest priorities of preventive health-care services for children. Therefore, systematic vaccinations were distinguished from those that were non-systematic, and sometimes even controversial. These registers in the protocols also contained detailed information that allows us to detect those visits related to advice for some vaccinations that were non-systematic and controversial.

These three data sets were complementary in the sense that they allowed us to merge systematic vaccination registrations, preventive visits and population administrative health-care use data. To be included in the data set, individuals must have had at least one point of contact with the health-care system. Given the time span we analysed, it is mostly likely that more than 90% were covered through our data set. In any case, those who only used private health care to take care of their children were not addressed by this research project.

As regards SES, it was separately proxied by means of educational attainment levels and inferred salary records through the use of average earnings based on occupational codes. Average salary records in 2002 by occupational category were also provided by the Catalonian Statistics Institute. Mother’s education and father’s education have usually been considered separately because it is mostly the mother who takes care of children’s health, especially at younger ages [22]. Maternal employment status was also included.

Therefore, descriptive statistics for children and parents were calculated according to four different groups: personal characteristics; health status; health-care use; and lifestyle behaviour.

## Results

The final panel data set containing information about all four different linked databases comprised 11,415 individuals, corresponding to 79,905 observations. Descriptive statistics regarding the population analysed, both children and parents, are given in Tables 1 and 2, respectively. Table 3 shows the average number of child visits by parental educational level. Tables 1, 2, and 3 exhibit data from the first database. All means refer to the period studied.

**Table 1:** Descriptive statistics for children (*n* = 79,905 observations, corresponding to 11,415 individuals) for the period 2004–2012

*Personal characteristics*	Mean (SD)	*Health status*	Mean (SD)
Being female	0.484 (0.50)		
Patient’s age	6.270 (4.93)	No. different diseases in a year	2.095 (2.39)
Immigrant status	0.006 (0.08)	Charlson index	0.018 (0.15)
Minimum distance to GP	4.020 (4.51)	Hypertension	0.001 (0.03)
Health district ABS01	0.105 (0.31)	Lipid & cholesterol problems	0.006 (0.07)
Health district ABS03	0.147 (0.35)	Children with any cardio problem	0.006 (0.08)
Health district ABS08	0.132 (0.34)	Stroke/cerebrovascular accident	0.000 (0.01)
Health district ABS09	0.181 (0.39)	Bronchial asthma & obstructive pulmonary	0.070 (0.26)
Health district ABS10	0.125 (0.33)	Children with any mental disorder	0.002 (0.04)
Health district ABS12	0.119 (0.32)	Malignant neoplasms	0.002 (0.04)
Health district ABS15	0.192 (0.39)		
*Health-care use*	Mean (SD)	*Lifestyle behaviour*	Mean (SD)
Number of visits to GP	6.954 (8.68)		
Number of visits to specialists	0.394 (1.17)		
Number of hospitalized days	0.003 (0.07)	Median BMI	17.302 (3.22)
Number of urgent visits	0.087 (0.36)	Mean BMI	17.417 (3.12)
Number of laboratory tests	0.117 (0.44)	Smoker	0.000 (0.02)
Number of radiology tests	0.142 (0.49)	Alcoholism	0.000 (0.01)
Number of diagnostic tests	0.021 (0.17)		

Average values and standard deviations in brackets are reported. Descriptive statistics are reported for the whole considered period. Time distances (in minutes) were computed accounting for children’s residence and GP or hospital location by means of *geocode* and *traveltime* commands in Stata

Table 1 shows the descriptive statistics for children in terms of the personal characteristics, health status, health-care use, and lifestyle behaviour

**Table 2:** Descriptive statistics of parental information for the period 2004–2012

*Personal characteristics*	Mean (SD)	*Health status*	Mean (SD)
Father’s age	45.038 (9.05)	Parental consumption of medicines	0.412 (0.84)
Mother’s age	43.070 (8.79)	Father’s co-morbidity episodes	2.843 (2.05)
Parents are separated or divorced	0.046 (0.21)	Mother’s co-morbidity episodes	3.661 (2.40)
Father deceased	0.012 (0.11)	Father’s Charlson index	0.202 (0.60)
Mother deceased	0.004 (0.06)	Mother’s Charlson index	0.160 (0.50)
Father’s schooling years	9.819 (4.03)	Father’s mental problems	0.057 (0.23)
Mother’s schooling years	9.988 (4.14)	Mother’s mental problems	0.135 (0.34)
Maternal employment	0.669 (0.47)		
*Health-care use*	Mean (SD)	*Lifestyle behaviour*	Mean (SD)
Father’s visits to GP	8.188 (8.38)		
Mother’s visits to GP	10.261 (9.19)	Father’s average BMI	27.693 (4.25)
Father’s hospitalization days	0.500 (2.24)	Mother’s average BMI	26.253 (5.37)
Mother’s hospitalization days	0.318 (1.50)	Father smoker	0.275 (0.45)
Father’s urgent visits	0.670 (0.98)	Mother smoker	0.236 (0.42)
Mother’s urgent visits	0.667 (0.98)	Father’s alcoholism	0.027 (0.16)
Father’s visits to specialist	3.235 (4.42)	Mother’s alcoholism	0.004 (0.06)
Mother’s visits to specialist	3.486 (4.92)		

Average values and standard deviations in brackets are reported. Descriptive statistics are reported for the whole considered period. Time distances (in minutes) were computed accounting for parents’ residence and GP or hospital location by means of *geocode* and *traveltime* commands in Stata

Table 2 shows the descriptive statistics for children’s parents in terms of the personal characteristics, health status, health-care use, and lifestyle behaviour

**Table 3:** Average number of child visits by parental educational level

		Mean visits to GP	Mean visits to specialists	Mean emergencies
Fathers	Illiterate	7.78	0.39	0.10
	Primary	7.38	0.42	0.10
	Secondary	7.40	0.46	0.10
	Vocational	7.45	0.46	0.10
	Upper secondary	6.80	0.36	0.08
	3-year degree	5.86	0.29	0.06
	Higher education	5.23	0.25	0.06
	Post-higher education	4.92	0.16	0.07
Mothers	Illiterate	7.33	0.36	0.10
	Primary	7.50	0.44	0.11
	Secondary	7.55	0.48	0.11
	Vocational	7.31	0.44	0.10
	Upper secondary	6.61	0.35	0.07
	3-year degree	6.21	0.29	0.06
	Higher education	5.55	0.26	0.06
	Post-higher education	5.08	0.18	0.09

Table 3 shows the average number of child visits by father’s and mother’s educational level, according to the classification of: illiterate; primary school; secondary school; vocational; upper secondary school; 3-year degree; higher education; and post-higher education

Table 1 shows that the average number of visits to GPs and specialists were 6.95 (SE 8.68) and 0.39 (SE 1.17), respectively. In terms of health status, the average number of different diseases in a year was approximately 2.1 (SE 2.39) with a Charlson co-morbidity index of 0.018 (SE 0.15), With respect to parents, the average number of fathers’ schooling years was 9.82 (SE 4.03) while for mothers it was 9.99 (SE 4.14). In terms of the health case use, the average number of visits to GPs and specialists was higher for mothers than for fathers, whereas for urgent visits and for hospitalization days it was slightly higher for fathers than for mothers. Overall, the parental consumption of medicines was 41.2% (SE 0.84), with on average more co-morbidity episodes for mothers (3.66) than for fathers (2.84), although the Charlson co-morbidity index was higher for fathers (0.20) than for mothers (0.16), indicating that the latter suffered more co-morbidities than fathers. The results in Tables 1 and 2 have wide standard errors for many of the parameters, thereby showing the typical behaviour of administrative data. Table 3 shows the average number of child visits, in general, by parental education level. This table clearly shows that the higher the education level of parents, the lower the number of GP visits.

Results regarding the influence of education on the childhood immunization schedule within the “Healthy Child Programme” and the visits for vaccination information are shown in Table 4.

**Table 4:** Healthy Child Programme and visits for vaccination: negative binomial regressions

	Up to 1 year old	1–2	2–3	3–6	6–8	8–12	12–16	Visits for advice about systematic vaccinations	Visits for advice about controversial vaccinations
	Coef (s.e.)	Coef (s.e.)	Coef (s.e.)	Coef (s.e.)	Coef (s.e.)	Coef (s.e.)	Coef (s.e.)	Coef (s.e.)	Coef (s.e.)
Father’s schooling years	−0.04^a^ (0.009)	− 0.03^a^ (0.008)	− 0.03^a^ (0.008)	− 0.02^b^ (0.007)	− 0.009 (0.009)	0.004 (0.009)	− 0.03^c^ (0.01)	0.03^c^ (0.01)	0.06^b^ (0.02)
Mother’s schooling years	− 0.04^a^ (0.009)	− 0.03^a^ (0.009)	− 0.03^a^ (0.008)	− 0.02^a^ (0.007)	− 0.01 (0.009)	0.02 (0.009)	0.009 (0.01)	−0.008 (0.01)	− 0.03 (0.02)
Inferred salaries at household level	−0.009^a^ (0.002)	− 0.009^a^ (0.002)	− 0.005^a^ (0.002)	− 0.005^a^ (0.001)	−0.006^a^ (0.002)	− 0.003^c^ (0.002)	−0.002 (0.002)	0.006^b^ (0.002)	0.004 (0.004)
Maternal employment	0.2^c^ (0.08)	0.2^b^ (0.08)	0.3^a^ (0.07)	0.5^a^ (0.06)	0.7^a^ (0.07)	0.3^a^ (0.07)	−0.02 (0.09)	0.1 (0.1)	0.2 (0.2)
Child characteristics	included	included	included	included	included	included	included	included	included
Parental information	included	included	included	included	included	included	included	included	included
Sample size	4557	5219	5876	7894	5753	6509	3879	50,506	50,397
χ^2^	369.8 (0.00)	280.4 (0.00)	120 (0.00)	762 (0.00)	949.5 (0.00)	1706.9 (0.00)	664.1 (0.00)	33.9 (0.00)	18.2 (0.00)
Pseudo-R^2^	0.06	0.04	0.02	0.07	0.1	0.2	0.1	0.005	0.007

Standard errors are reported in brackets, whereas ^a^, ^b^, and ^c^ denote significance levels of 1, 5, and 10%, respectively. Regressions include a dummy variable representing odd minimum distance. Children’s chronic conditions include the following diseases: hypertension, lipid & cholesterol problems, children with any cardio problem, stroke/cerebrovascular accident, bronchial asthma, chronic obstructive pulmonary disease, children with any mental disorder and malignant neoplasms. We also included unhealthy behaviours, which are relevant for adolescents (smoking and heavy drinking). Parental information includes information either with regard to fathers or mothers: age, any parent having passed away, parental consumption of medicines in thousand euros, co-morbidity episodes, mental problems, maternal employment, unhealthy behaviours (smoking and heavy drinking), health-care use (visits to GP, specialists, emergencies, and hospitalization)

Table 4 shows the results regarding the influence of education on the childhood immunization schedule within the “Healthy Child Programme” and the visits for vaccination information. The first seven columns show different age intervals because in each of these the vaccination protocol was different. The last two columns refer to the average number of visits for advice in terms of vaccination, both systematic and controversial. This table describes results from databases 1 and 2 merged

The first seven columns of Table 4 show different age intervals because in each of these the vaccination protocol was different. The last two columns refer to the average number of visits for advice in terms of vaccination, both systematic and controversial. This table presents the results from databases 1 and 2 merged. As can be seen, the higher the educational attainment level of the parents, the lower the number of visits for this public health programme.

Lastly, in terms of vaccination registers, the results are shown in Table 5, which displays the likelihood of being vaccinated and the mean number of vaccinations according to the children’s age.

**Table 5:** Vaccinations registers: logistic and negative binomial regressions

	Likelihood of being vaccinated	Likelihood of being vaccinated against human papillomavirus	Number of visits for vaccinations	Number of vaccinations	No. of vaccinations age 0–6	No. of vaccinations age 6–16
	Coef (s.e.)	Coef (s.e.)	Coef (s.e.)	Coef (s.e.)	Coef (s.e.)	Coef (s.e.)
Father’s schooling years	−0.001 (0.01)	− 0.000 (0.01)	− 0.006 (0.00)^a^	− 0.006 (0.00)^a^	− 0.012 (0.00)^a^	0.000 (0.00)
Mother’s schooling years	− 0.000 (0.01)	0.005 (0.00)^a^	− 0.007 (0.00)^a^	− 0.007 (0.00)^a^	− 0.013 (0.00)^a^	0.010 (0.00)^b^
Inferred salaries at household level	− 0.0003 (0.00)^b^	0.001 (0.00)^a^	− 0.002 (0.00)^a^	− 0.002 (0.00)^a^	− 0.003 (0.00)^a^	0.001 (0.00)^c^
Child characteristics	included	included	included	included	included	included
Parental information	included	included	included	included	included	included
Sample size	6415	5525	6415	6415	4830	1585
χ^2^	2226.52 (0.00)	1760.64 (0.00)	943.84 (0.00)	980.15 (0.00)	620.47 (0.00)	1220.38
Pseudo-R^2^	0.3205	0.2528	0.0153	0.0164	0.0074	0.0212

Standard errors are reported in brackets, whereas ^a^, ^b^, and ^c^ denote significance levels of 1, 5, and 10%, respectively. Regressions include a dummy variable representing odd minimum distance. Children’s chronic conditions include the following diseases: hypertension, lipid & cholesterol problems, children with any cardio problem, stroke/cerebrovascular accident, bronchial asthma, chronic obstructive pulmonary disease, children with any mental disorder and malignant neoplasms. We also included unhealthy behaviours, which are relevant for adolescents (smoking and heavy drinking). Parental information includes information either with regard to fathers or mothers: age, any parent having passed away, parental consumption of medicines in thousand euros, co-morbidity episodes, mental problems, maternal employment, unhealthy behaviours (smoing and heavy drinking), health-care use (visits to GP, specialists, emergencies, and hospitalization)

Table 5 displays the likelihood of being vaccinated and the mean number of vaccinations according to the children’s age

There was no statistical significance for the likelihood of being vaccinated. In terms of number of visits, the higher the parental education, the lower the number of visits and vaccinations. In addition, the issue of being immunized against the human papillomavirus was explored. The results showed that the higher the mother’s educational attainment level, the greater the probability of being vaccinated for this specific immunization. Finally, the presence of an age profile for vaccinations showed that less educated parents visited GPs more frequently for immunizations when their children were below the age of six, but that pattern was completely the opposite after that age. Hence, for children aged between six and 16, more educated parents were more likely to ensure that their children were immunized. Likewise, systematic vaccinations were more likely for those parents with a lower educational attainment level.

## Discussion

This paper evidenced the presence of an education gradient for preventive care through the public health system. More educated parents were more likely to make preventive recorded GP visits without any particular disease and more prone to visit GPs to get advice about systematic or non-systematic controversial vaccinations. The education gradient was analysed in a publicly funded health-care system with universal coverage for children. The use of administrative data for counts constitutes in itself a contribution to this literature since most of the studies rely on defining health status by means of self-assessed measures (health status and chronic conditions).

From the results can be inferred that the higher the educational attainment level of the parents, the lower the number of visits for this public health programme. This was an expected finding given that the programme is addressed to children at greater risk and calls were made to ensure their attendance. Nevertheless, for the last three age categories (columns 5 to 7) no statistical significance was found. This finding is contrary to the above-mentioned age profile for the total number of visits but this might be related to the fact that calls were made more frequently for attendance at early ages. Those visits, being a consequence of the “Healthy Child Programme”, confirmed the purpose of the programme and generated more visits from riskier households. Irrespectively of the nature of the provided vaccinations, we evidence a positive association between parental schooling years and the likelihood of these visits for advice with regard to vaccinations.

It was important to separate the role of education from income gradient effects. The better educated are less likely to experience risky behaviours (smoking, drinking heavily, being overweight, or using illegal drugs) due to heterogeneity in preferences or in discount rates. More preventive care, greater effectiveness in managing children’s chronic health conditions, taking more advantage of new medical technologies, and better access to, or assimilation of, health information [23] are all expected when a higher level of parental education is attained. The existence of an SES gradient in children’s health is extensively documented in developed countries [13] no matter which specifically SES dimension was considered (income, wealth, or education). On the one hand, two pioneering and influential papers evidenced this association for the SES income gradient. Some authors evidenced the influence of parents’ income on improving children’s health as children aged [13]. The latter was also found for Canada [24]. More recently, several papers have corroborated this positive association between household income and children’s health for the US [25], the UK [26], Germany [15], and Australia [27], although the last of these shows a smaller income gradient than the other countries which have universal health-care financing insurance.

So far, after the use of more objective measures, the income gradient has not been evidenced [26]. To the best of our knowledge, no previous literature has addressed the specific question regarding the extent to which the misreporting of children’s health by parents is driven by their socio-economic status, but, a priori, a similar education gradient would be expected. In this regard, there was evidence of maternal misreporting of their children’s health by comparing their assessments with those of their partners, although they did not show the underpinning reasons.

This study also presents some limitations. In Catalonia a great part of the population has duplicate health care coverage, public and private, most in the privileged population. This might influence the conclusions of the study. Private practitioners may administrate freely all vaccines but not HPV in the adolescence, and this could explain part of the effect of SES. The use of three different databases constitutes a limitation when encoding. Likewise, although there is significant knowledge on the impact of parental education on vaccination delay and immunization status in developed countries, little is known when we address education gradient in differentiating between systematic and non-systematic vaccinations. However, compared to previous research, this is the first work to use administrative data and explore factors conditional on visits for advice. The demographic and economic data on parents from 2002 may have changed in the last 15 years, and no dynamicity in the update of socio-economic characteristics has been contemplated. Therefore, the level of education might have changed over the years contemplated in the analysis, and therefore the results might not reflect the current situation. This is one of the characteristics of using longitudinal data, which are static by their very nature and end with a loss to follow-up over time. However, a static analysis could be just enough to study the influence, at a given point in time, of education over children’s immunization programmes.

These findings can be used to inform policy and practice to help information and educational campaigns move forward in terms of systematic and non-systematic vaccinations of children.

## Conclusion

The use of administrative longitudinal information in Catalonia (Spain) has evidenced that more educated parents are more likely to make preventive recorded GP visits without any particular disease and were more prone to visit GPs to get advice about systematic or non-systematic controversial vaccinations. On the other hand, those visits, being a consequence of a specific children’s vaccination programme, generated more visits from riskier households. Likewise, systematic vaccinations were more likely for those parents with a lower educational attainment level.

## Abbreviations

ECDC: European Centre for Disease Prevention and Control
EU: European Union
GP: General practitioner
HCU: Children’s health-care use
ICPC-2: International classification of primary care, second edition
SAP: Spanish Association of Paediatrics
SES: Socio-economic status
